# Winter forage crops influence soil properties through establishing different arbuscular mycorrhizal fungi communities in paddy field

**DOI:** 10.1007/s44307-024-00037-5

**Published:** 2024-09-09

**Authors:** Mengyan Cao, Yao Xiang, Lingyue Huang, Menghao Li, Cheng Jin, Chuntao He, Guorong Xin

**Affiliations:** State Key Laboratory of Biocontrol, Guangdong Provincial Key Laboratory of Plant Stress Biology, School of Agriculture and Biotechnology, Shenzhen Campus of Sun Yat-Sen University, Sun Yat-Sen University, Shenzhen, 518107 Guangdong China

**Keywords:** Winter forage crops, Arbuscular mycorrhizal fungi, Soil physicochemical properties, Paddy field ecosystem

## Abstract

Winter planting is promising for improving the utilization rate of fallow paddy fields in southern China by establishing arbuscular mycorrhizal fungi (AMF) communities. However, the effects of different winter forage crops on AMF community construction remain unknown. The AMF community establishment of different winter planting forage crops were conducted in oat, rye, Chinese milk vetch, and ryegrass, with winter fallow as a control. The AMF colonization rate, soil AMF spore density, community structure and diversity, and soil physicochemical properties were determined. The results showed that the total nitrogen and available nitrogen in winter Chinese milk vetch were 11.11% and 16.92% higher than those in winter fallow (*P* < 0.05). After planting winter forage crops, the AMF spore density in winter oat, rye, Chinese milk vetch, and ryegrass soil were 127.90%, 64.37%, 59.91%, and 73.62% higher than that before planting, respectively (*P* < 0.05). *Claroideoglomus* was the dominant AMF genus in the soil of winter planting oat, rye, and ryegrass. The average membership function value of winter Chinese milk vetch was the highest, indicating that it had the best comprehensive effect on soil physicochemical properties, AMF community structure and diversity, and fresh forage yield. Winter forage crops could increase the spore pool of soil AMF and improve the soil AMF community structure and diversity. Winter Chinese milk vetch in paddy field had the best comprehensive effect on soil physicochemical properties and soil AMF community according to the comprehensive evaluation. These findings provide a theoretical basis for sustainable development and utilization of the southern rice paddy ecosystem.

## Introduction

Arbuscular mycorrhizal fungi (AMF) are endosymbiotic fungi capable of forming stable symbiotic structures in most terrestrial plants (Smith and Read 2008). The mycorrhizal structure formed by AMF and plant roots can promote the growth, development, nutrient uptake, and stress resistance of host plants (Neumann and George 2010; Orlowska et al. 2012; Estrada et al. 2013). The ecological functions of the soil AMF community structure and diversity include the improvement of plant community stability and plant system productivity (Yang et al. 2016). When the AMF community diversity is low, the plant community structure and composition are unstable (Maherali et al. 2007). An increase in AMF community diversity can reduce competition between legumes and herbaceous plants and promote community stability (Wagg et al. 2011). With the increase of AMF species diversity, the corresponding functional diversity also increases, and the productivity of plant community increases through "functional complementarity" (Vogelsang et al. 2006; Wagg et al. 2011). Because AMF are symbiotic microorganisms in plant roots, host plant species determine AMF diversity to a certain extent (Aidee et al. 2021). In flooded paddy fields, the AMF colonization rate is low owing to a lack of oxygen, but AMF spores can be preserved in the soil (Lumini et al. 2011), which would contribute to the improvement of rice production (Cao et al. 2024). Few studies have investigated the effects of planting different crops on AMF communities in paddy soils during the winter fallow period.

There are abundant fallow paddy fields in southern China during winter that are suitable for crop growth with sufficient light, water, and heat resources (Wu et al. 2018). After long-term continuous double cropping rice in southern China, the soil is prone to problems such as compaction and deterioration of its physical and chemical properties (Gao et al. 2013). In the absence of vegetation cover, the soil is more susceptible to erosion, which accelerates the leaching of various elements (Xin et al. 2011). The introduction of crop rotation systems in winter fallow paddy fields can effectively improve soil conditions and avoid continuous cropping obstacles. Winter planting of green manure crops in paddy fields not only make effective use of winter fallow rice fields but also improve soil physicochemical properties and microbial communities, and produce a large amount of high-quality green feed to alleviate the shortage of forage crops during winter in southern China (Huang et al. 2022).

Common paddy rotation systems in southern China are generally constructed by the forage crops such as "rice-rice-Chinese milk vetch" and "rice-rice-ryegrass". Italian ryegrass (*Lolium multiflorum* L.) is widely cultivated in winter paddy fields to provide sufficient fodder (Yang et al. 2008; Tao et al. 2017), which can alleviate soil acidification and increase soil organic matter content (He et al. 2019), soil porosity, and soil water retention capacity (Madari et al. 2005; Qiao et al. 2017). Chinese milk vetch (*Astragalus sinicus* L.) is a legume green manure with high nutritional content and good quality that serves as a forage crop (Liu et al. 2020a), which can be symbiotic with rhizobia for biological nitrogen fixation to supplement soil nitrogen reservoirs (Li et al. 2019b). Similarly, other forage crops, such as oat (*Avena sativa* L.) and rye (*Secale cereale* L.), have become the dominant crops to be popularized and planted in southern winter fallow fields because of their cold resistance, strong adaptability, high yield, and high feeding value characteristics (Wang et al. 2016; Ma et al. 2020). Compared with cash crops, these forage crops not only provide a large amount of green fodder during winter, but can also be returned to the field as green manure, increasing the soil nutrient content and thus reducing fertilizer application (Zhang et al. 2017; Xu et al. 2021). The composition and diversity of the AMF community in the soil were affected by the different forage treatments. The relative abundances of *Acaulospora* and *Diversispora* increased significantly in soil planted with alfalfa (*Medicago sativa* L.) for a long time (Pellegrino et al. 2019). Legume nitrogen fixation can affect the soil AMF community composition (Xiao et al. 2019). *Acaulospora* and *Septoglomus* have high colonization rates and adaptability to leguminous forage-growing areas (Xiao et al. 2020). Planting ryegrass increased the AMF colonization rate and AMF community richness and increased the relative abundance of soil Glomerales and Archaeosporales (Garcia-González et al. 2023). However, few studies have focused on AMF communities from the winter planting of different forage crops in paddy fields.

The influences of different forage crops on AMF communities in winter paddy fields in southern China were explored using winter fallow, winter ryegrass, winter Chinese milk vetch, winter rye, and winter oat. Our hypotheses were as follows: (1) Winter planting of different forage crops has distinct effects on soil physicochemical properties. (2) Winter planting of forage crops can increase the AMF community diversity in paddy fields. (3) Different forage crops domesticate different AMF communities in the paddy fields.

## Materials and methods

### Experimental site description and design

The field experiment was conducted in Ai-rice Daoxiang Town (23°55’N, 113°56’E), Conghua District, Guangzhou, Guangdong Province, from November 2021 to March 2022. The experimental site was located approximately 35 m above sea level and had a subtropical monsoon climate. The average monthly precipitation during the experiment was only 42.4 mm. The highest temperature during the experiment period was 23 °C and the lowest temperature was 9 °C. The soil type was red soil, the paddy fields were planted with rice for two seasons per year before the experiment began, and field fallow in winter. The soil fertility at the experimental site was uniform. The soil background pH was 5.71 and soil organic matter (SOM), total nitrogen (TN), total phosphorus (TP), total potassium (TK), available nitrogen (AN), available phosphorus (AP), and available potassium (AK) were 23.14 g/kg, 1.32 g/kg, 0.67 g/kg, 18.52 g/kg, 102.15 mg/kg, 55.67 mg/kg, and 110.01 mg/kg, respectively.

Four forage crops suitable for winter planting in southern China were selected: *Astragalus sinicus* L. (MV), *Avena sativa* L. (O), *Secale cereale* L. (R), and *Lolium multiflorum* L. (RG). The sowing amount was 25 kg/ha. A random block design was adopted; each plot had an area of 20 m^2^ and three replicates of each crop were planted. The winter fallow treatment was performed using the same number of repeats. The winter forage crops were sown on November 15, 2021, and harvested on March 30, 2022. No fertilizer was applied during planting. The AMF colonization rate was determined by collecting root samples from plants in the crop harvest period. The soil samples were collected using a five-point sampling method. Each repeated soil sample was divided into three parts. A portion of the soil was air-dried and screened to 0.149 mm for the determination of the soil physical and chemical properties. A part of samples was stored in -80 ℃ refrigerator for soil AMF community structure and diversity analysis. The other part of the soil samples was temporarily stored in -20 ℃ refrigerator to count the AMF spore density in the soil.

### Determination of soil chemical properties and forage yield

Soil pH was determined using a pH meter (PE-10; Sartorious, Germany) with a fresh soil to water ratio of 1:2.5 (w/v). Soil organic matter (SOM) was measured by combustion using a CNS elemental analyzer (CNS-2000; LECO, St. Joseph, MI, USA). Soil total nitrogen (TN) was measured using the Kjeldahl digestion method, soil total phosphorus (TP) was measured using molybdenum-antimony resistance colorimetry, and flame photometry was used to determine soil total potassium (TK) (Bao 2000). Available nitrogen (AN) was measured using the alkaline hydrolysis diffusion method with 1 mol/L NaOH, soil available phosphorus (AP) was determined by HCl-H_2_SO_4_ digestion, and soil available potassium (AK) was determined by NH_4_OA_c_ solution extraction and flame photometry (Lu 2000).

During forage harvesting, five 20 cm × 20 cm quadrats were selected in each plot, all forage ground parts in the quadrat were harvested, fresh weight was measured, and yield was calculated. The collected fresh grass was dried for 1 h at 105℃, and then at 75℃ to constant weight.

### Determination of AMF colonization rate and spore density

Fresh plant roots were selected and cut into 1 cm long root segments. After clearing with KOH, the roots were stained with trypan blue (Philips and Hayman 1970). Stained root segments were mounted on slides and examined under a microscope. The mycorrhizal colonization rate was calculated as the number of fields with mycorrhiza/total root segments × 100% (Cheng et al. 2019). 10 g of dried soil were weighed for each replicate. The AMF spores were separated from the 10 g soil by wet sieve decanting and sucrose density gradient centrifugation through 0.8 mm, 0.25 mm, and 0.045 mm screens from top to bottom, and then counted under a dissecting microscope (Trejo-Aguilar and Banuelos 2020).

### DNA extraction and sequencing of soil AMF community

Soil DNA was extracted using an E.Z.N.A.® soil Kit (Omega Bio-tek, Norcross, GA, U.S.). DNA concentration and purity were measured by NanoDrop2000 UV–vis spectrophotometer (Thermo Scientific, Wilmington, DE, USA), and the quality of the extracted DNA was measured using 1% agarose gel electrophoresis. The fungal 18S rDNA gene was amplified by nested PCR, and AML1(5'-ATCAACTTTCGATGGTAGGATAGA-3') and AML2(5'-GAACCCAAACACTTTGGTTTCC-3') were selected as the first pair of primers. The amplified fragment was approximately 800 bp. AMV4-5NF (5'-AAGCTCGTAGTTGAATTTCG-3') and AMDGR (5'-CCCAACTATCCCTATTAATCAT-3') served as the second pair of primers-amplified fragments of about 300 bp (Tian et al. 2021). In the second round of PCR, Barcode was added to the primers AMV4-5NF and AMDGR for sample differentiation. The PCR products were extracted using a 2% agarose gel and purified using an AxyPrep DNA gel extraction kit (Axygen Biosciences, Union City, CA, USA). The purified PCR products were QuantiFluor™-ST (Promega, USA). These amplifications were subsequently combined and sequenced on the Illumina MiSeq platform in equal molar quantities (Zhang et al. 2020).

### Applying fuzzy membership function to comprehensive evaluation

The fuzzy membership function was used to evaluate the effects of different treatments on the soil physicochemical properties and AMF communities (Wang et al. 2023). The membership function value of a certain index for each treatment was calculated, and then the average membership function value for each treatment was calculated. A larger value indicates a higher comprehensive evaluation of the treatment. The calculation method is as follows:

1 $${U}_{ij}=\frac{{X}_{ij}-{X}_{i\text{min}}}{{X}_{i\text{max}}-{X}_{i\text{min}}}$$

2 $$U_i=\frac1n{\textstyle\sum_{j=1}^n}U_{ij}$$

*U*_*ij*_ is the membership function value of *j* index in *i* treatment, *X*_*ij*_ is the average value of *j* index in *i* treatment, *X*_*imax*_ is the maximum value of this index in all treatments. *X*_*imin*_ is the minimum value of this index in all treatments. *U*_*i*_ is the average membership function value for each index in *i* treatment. If a certain index is negatively correlated with the variety selection goal, the membership function value is calculated using the inverse membership function method, as follows:

3 $${U}_{ij}=1-\frac{{X}_{ij}-{X}_{i\text{min}}}{{X}_{i\text{max}}-{X}_{i\text{min}}}$$

### Data analysis

The obtained sequencing data were assigned to different samples according to the barcode sequence, which was then removed. Quality control was performed before analyzing the raw data to obtain high-quality sequences. Cluster analysis was performed on the processed sequences. Operational taxonomic units (OTU) were divided by 97% similarity using UPARSE software. BLAST software was used to compare the OTU representative sequences (the most abundant sequences in each OTU as representative sequences) with the NCBI microbial species classification database (ftp://ftp.ncbi.nih.gov/pub/taxonomy/), and the species information with the highest identity value was selected as the species classification information of the OTU. The α diversity analysis (Chao, Shannon) and β diversity analysis of OTU were performed by QIIME software. R Studio V3.6.1 software to perform principal component analysis (principal co-ordinates analysis, PCoA; based on the Bray–Curtis distance algorithm), distance-based redundancy analysis (db-RDA), and correlation heat maps. R Studio V3.6.1 and Gephi V0.9.2 were used to map the correlation networks between the soil AMF communities. One-way analysis of variance (ANOVA) was performed using SPSS (version 23.0; IBM, Armonk, NY, USA). The least significant difference (LSD) was used to compare means at the 5% confidence level. Origin 2021 was used to draw the bar and box charts. Numerical values are the means ± standard errors.

## Results

### Effects of different winter forage crops on soil physicochemical properties

The physical and chemical properties of the soil varied among the forage crops planted in winter (Table 1). The soil pH in winter fallow was the highest (5.81) and was the lowest after winter planting rye (5.57). With the exception of winter oat, planting other winter forage crops reduced soil pH to different degrees without significant differences compared with the background soil (*P* > 0.05). The SOM content in winter Chinese milk vetch was the highest, 15.13% higher than that in the background soil (*P* < 0.05); however, the differences among the five treatments were not significant (*P* > 0.05). The soil TN content of winter Chinese milk vetch was 26.64 g/kg, which was significantly higher than that of the background, winter fallow, winter oat, and winter ryegrass (*P* < 0.05). The highest AN content was found in winter rye (111.55 mg/kg), which was significantly higher than that in winter fallow, winter oat, and winter ryegrass (*P* < 0.05). The soil TP content of winter Chinese milk vetch was the highest (0.72 g/kg), and that of winter oat was the lowest (0.62 g/kg). There were no significant differences among the TP contents of the other treatments (*P* > 0.05). The AP content in the background soil was the highest and decreased to different degrees after the treatments. The AP content in the winter oat soil was significantly lower than those in the other treatments (*P* < 0.05). The TK content in winter forage crops decreased and the TK content in winter oat soil was the lowest (16.43 g/kg). The soil AK content of winter ryegrass was 69.78 mg/kg, which was significantly lower than that of other treatments (*P* < 0.05).

**Table 1:** Variation of soil physicochemical properties in different treatments

Treatment	pH	Organic matter (g/kg)	Total nitrogen (g/kg)	Available nitrogen (mg/kg)	Total phosphorus (g/kg)	Available phosphorus (mg/kg)	Total potassium (g/kg)	Available potassium (mg/kg)
B	5.71 ± 0.05^abc^	23.14 ± 0.42^b^	1.32 ± 0.04^c^	102.15 ± 9.37^ab^	0.67 ± 0.05^ab^	55.67 ± 1.93^a^	18.52 ± 0.60^a^	110.01 ± 2.10^a^
F	5.81 ± 0.18^a^	24.14 ± 1.55^ab^	1.35 ± 0.08^bc^	87.36 ± 10.49^c^	0.70 ± 0.01^ab^	48.55 ± 0.48^b^	18.55 ± 0.21^a^	109.42 ± 12.88^a^
O	5.76 ± 0.01^ab^	24.39 ± 1.93^ab^	1.36 ± 0.08^bc^	92.74 ± 7.61^c^	0.62 ± 0.04^b^	39.33 ± 2.15^c^	16.43 ± 0.90^c^	91.20 ± 41.49^a^
R	5.57 ± 0.08^c^	26.04 ± 2.42^ab^	1.47 ± 0.11^ab^	111.55 ± 9.09^a^	0.65 ± 0.11^ab^	47.79 ± 6.61^b^	17.18 ± 0.33^bc^	90.59 ± 17.26^a^
MV	5.62 ± 0.03^bc^	26.64 ± 0.89^a^	1.50 ± 0.02^a^	102.14 ± 12.76^ab^	0.72 ± 0.02^a^	46.95 ± 3.77^b^	17.40 ± 0.11^b^	84.34 ± 24.62^a^
RG	5.62 ± 0.05^bc^	24.68 ± 1.13^ab^	1.36 ± 0.05^bc^	94.42 ± 2.33^bc^	0.70 ± 0.01^ab^	49.00 ± 2.34^b^	17.46 ± 0.04^b^	69.78 ± 0.51^b^

O: winter oat, R: winter rye, MV: winter Chinese milk vetch, RG: winter ryegrass

^a,b,c^Different lowercase letters indicate significant differences among the treatments in the same column (*P* < 0.05)

### Yield of different forage crops

The results for different forage yields are shown in Table 2. The fresh forage yield and dry forage yield of ryegrass were the highest at 54,533.79 kg/ha and 7918.33 kg/ha, respectively. The fresh forage yield of ryegrass was 29.55%, 71.42%, and 43.96% higher than those of oat, rye, and Chinese milk vetch, respectively (*P* < 0.05). From high to low, the fresh and dry forage yields of different forage types were ryegrass, oat, Chinese milk vetch, and rye, and the difference among the different forage types was significant (*P* < 0.05).

**Table 2:** Yield of different forage crops

Treatment	Fresh forage yield (kg/ha)	Dry forage yield (kg/ha)
O	42,094.24 ± 2266.24^b^	6361.21 ± 416.35^b^
R	31,812.54 ± 1089.53^d^	4813.42 ± 220.46^d^
MV	37,881.34 ± 1617.06^c^	5398.04 ± 183.67^c^
RG	54,533.79 ± 1936.35^a^	7918.33 ± 359.79^a^

O: winter oat, R: winter rye, MV: winter Chinese milk vetch, RG: winter ryegrass

^a,b,c,d^Different lowercase letters indicate significant differences among the treatments in the same column (*P* < 0.05)

### AMF colonization rate and soil AMF spore density of different winter forage crops

**Fig. 1:**
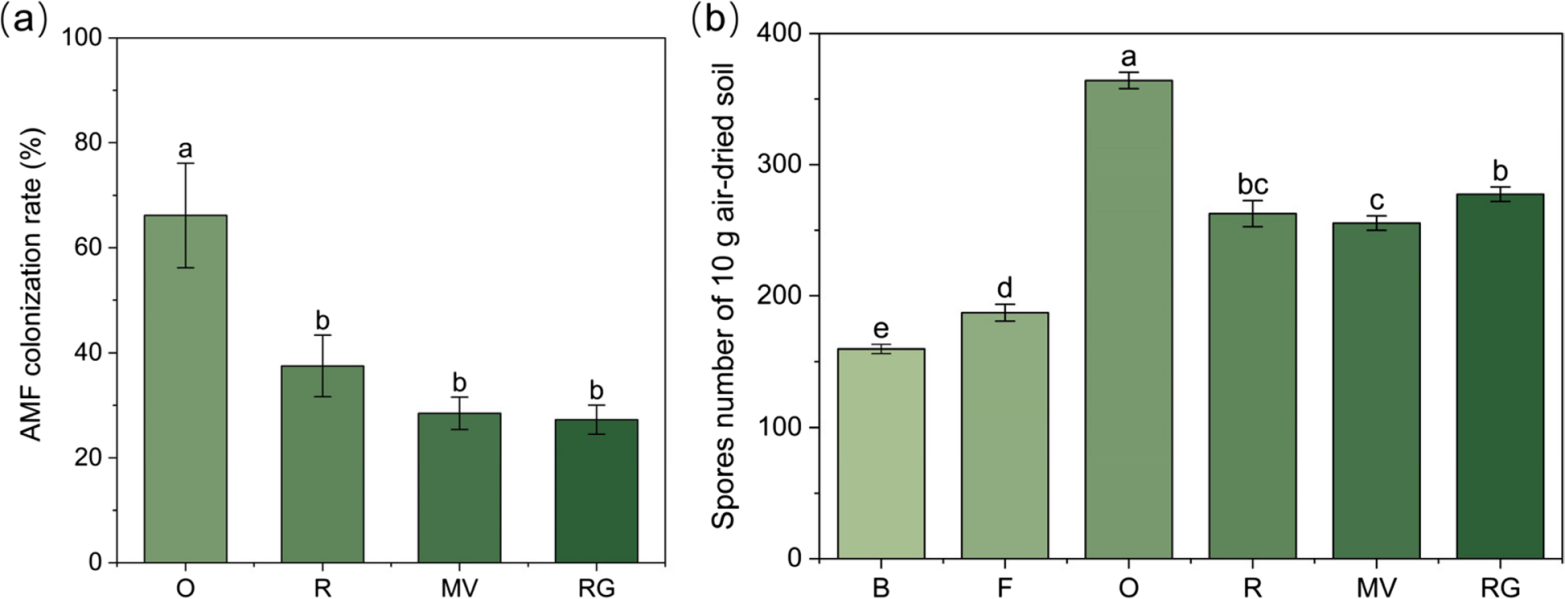
Characteristics of the arbuscular mycorrhizal fungi (AMF) communities in different winter forage crops. **a** AMF colonization rate. **b** Soil AMF spore density. Different lowercase letters indicate significant differences between the same indicators under different treatments (*P* < 0.05). B: background soil; F: winter fallow; O: winter oat; R: winter rye; MV: winter Chinese milk vetch; RG: winter ryegrass

The AMF colonization rates of different winter forage crops are shown in Fig. 1(a). The AMF colonization rate of oat was the highest (66.15%), and was significantly higher than those of rye, Chinese milk vetch, and ryegrass (*P* < 0.05). After winter forage crops, the soil AMF spore density increased to a certain extent (Fig. 1b). The AMF spore densities in winter oat, rye, Chinese milk vetch, and ryegrass soil were 127.90%, 64.37%, 59.91%, and 73.62% higher than that before planting, respectively (*P* < 0.05). The number of AMF spore density in the winter oat treatment reached 364.13, which was the highest among all the treatments.

### AMF community structure and diversity under different treatments

**Fig. 2:**
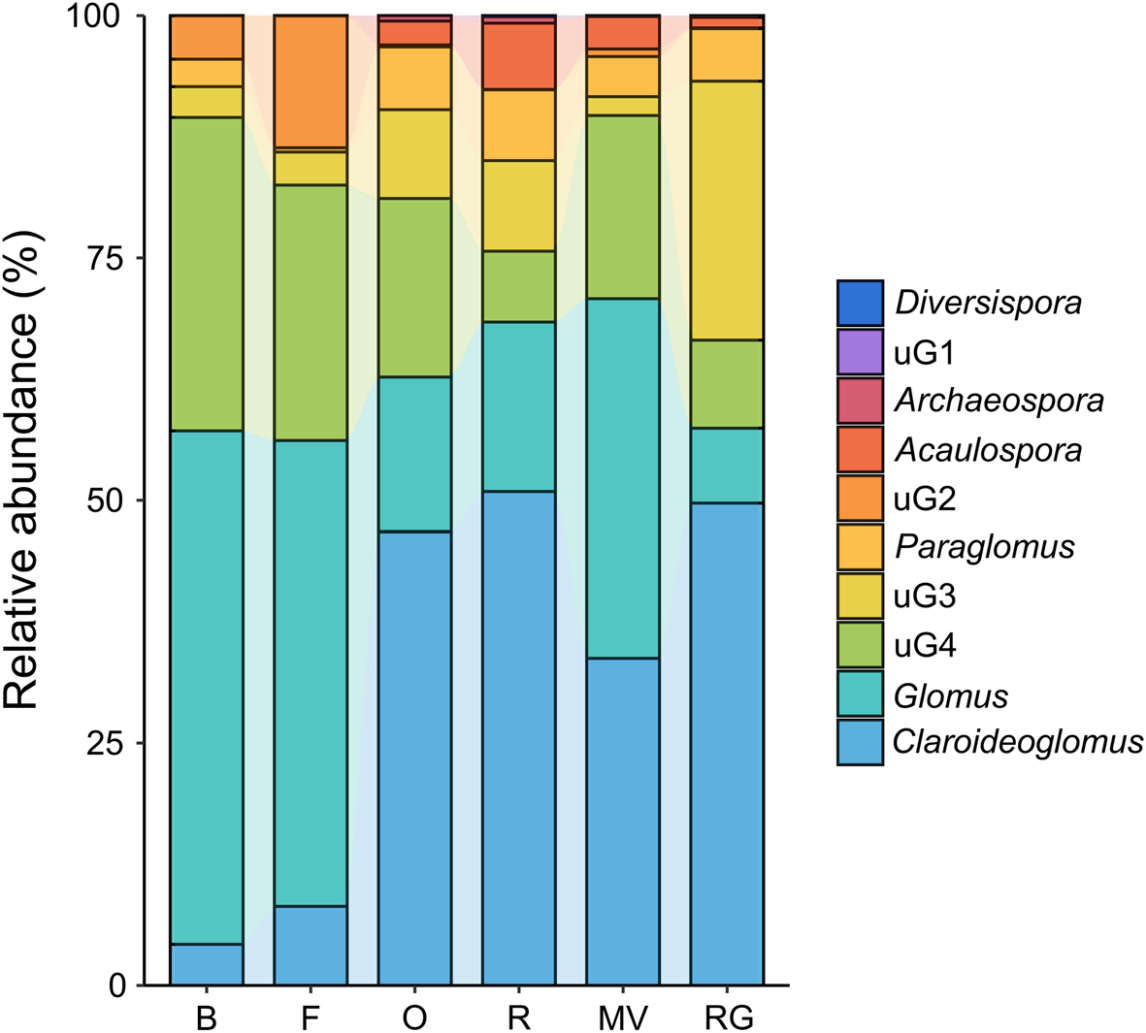
Composition of AMF community in different treatments. B: background soil; F: winter fallow; O: winter oat; R: winter rye; MV: winter Chinese milk vetch; RG: winter ryegrass. uG1: *unclassified_Gigasporaceae*; uG2: *unclassified_Glomeromycetes*; uG3: *unclassified_Glomeromycota*; uG4: *unclassified_Glomerales*.

The AMF community composition in the soil under different treatments is shown in Fig. 2. *Glomus* was the dominant genus in both background and winter fallow soils. After planting forage crops in winter, the soil AMF community composition changed with an increased relative abundance of *Claroideoglomus*, *Paraglomus* and *Acaulospora*. In the soil of winter oat, rye, and ryegrass, *Claroideoglomus* was the dominant genus, *Glomus* was the secondary dominant genera. The relative abundance of *Glomus* (37.05%) was the highest in the soil of winter Chinese milk vetch, but was not significantly different from that of *Claroideoglomus* (33.74%).

**Fig. 3:**
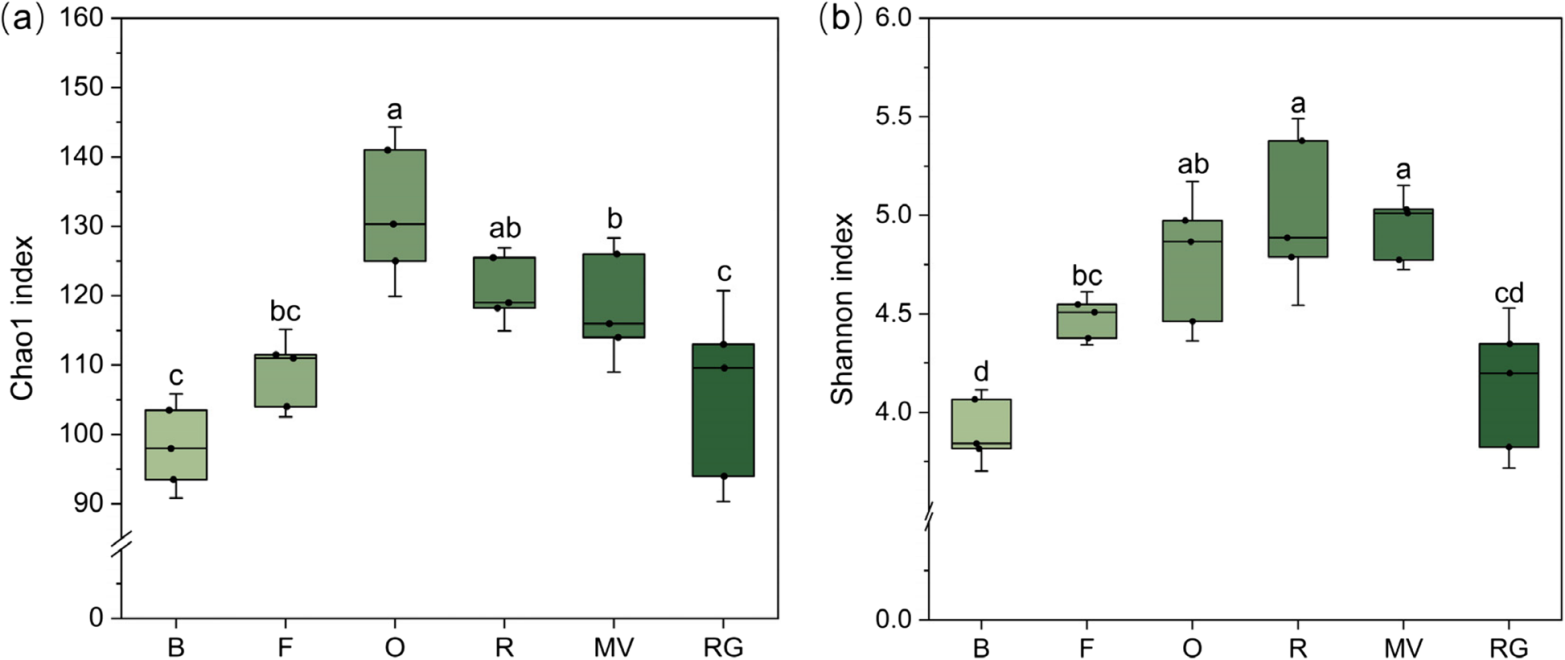
α-diversity index of AMF communities in different winter forage crops **a** Chao1 index of AMF community. **b** Shannon index of AMF community. Different lowercase letters indicate significant differences between the same indicators under different treatments (*P* < 0.05). B: background soil; F: winter fallow; O: winter oat; R: winter rye; MV: winter Chinese milk vetch; RG: winter ryegrass

**Fig. 4:**
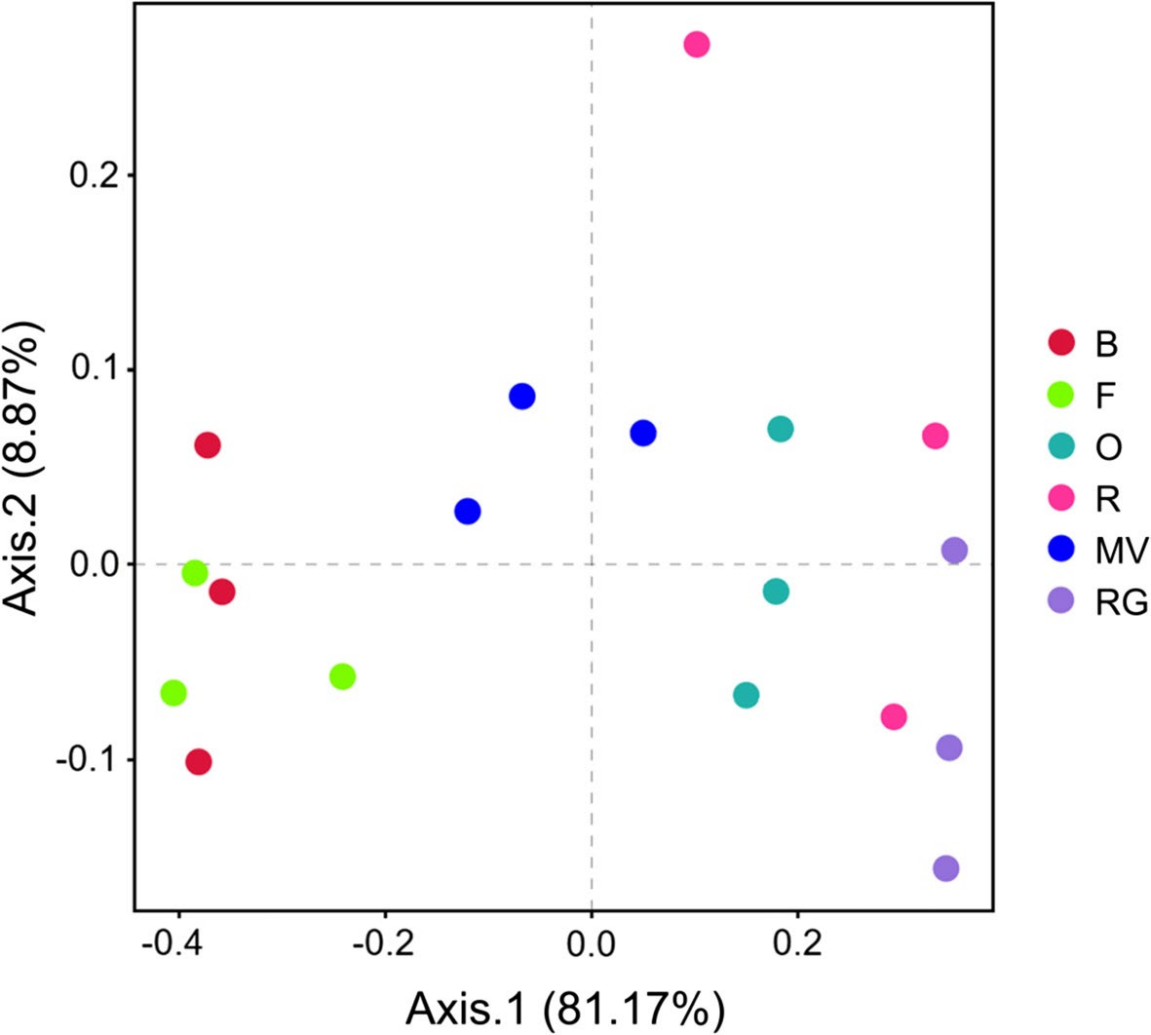
Principal co-ordinates analysis based on the Bray–Curtis distances showing the AMF communities on different treatments. B: background soil; F: winter fallow; O: winter oat; R: winter rye; MV: winter Chinese milk vetch; RG: winter ryegrass

The α diversity indices (Chao1 and Shannon indices) of AMF community under different treatments were shown in Fig. 3. The Chao1 index of the winter oat was the highest, and the Chao1 index of the background soil was the lowest. The Shannon index of winter rye was the highest; however, there was no significant difference among winter rye, winter oat, and winter Chinese milk vetch (*P* > 0.05). The Chao1 and Shannon indices of the winter ryegrass treatments were not significantly different from those of the background soil and winter fallow treatments (*P* > 0.05). The principal coordinate analysis profiled by Bray–Curtis distances illustrated a significant difference in the AMF community structure among the different treatments (Fig. 4). The AMF community structure of the background soil was similar to that of the winter fallow. The AMF community structure in winter Chinese milk vetch was different from that of the other treatments.

### Correlations between the AMF genera and environmental factors

**Fig. 5:**
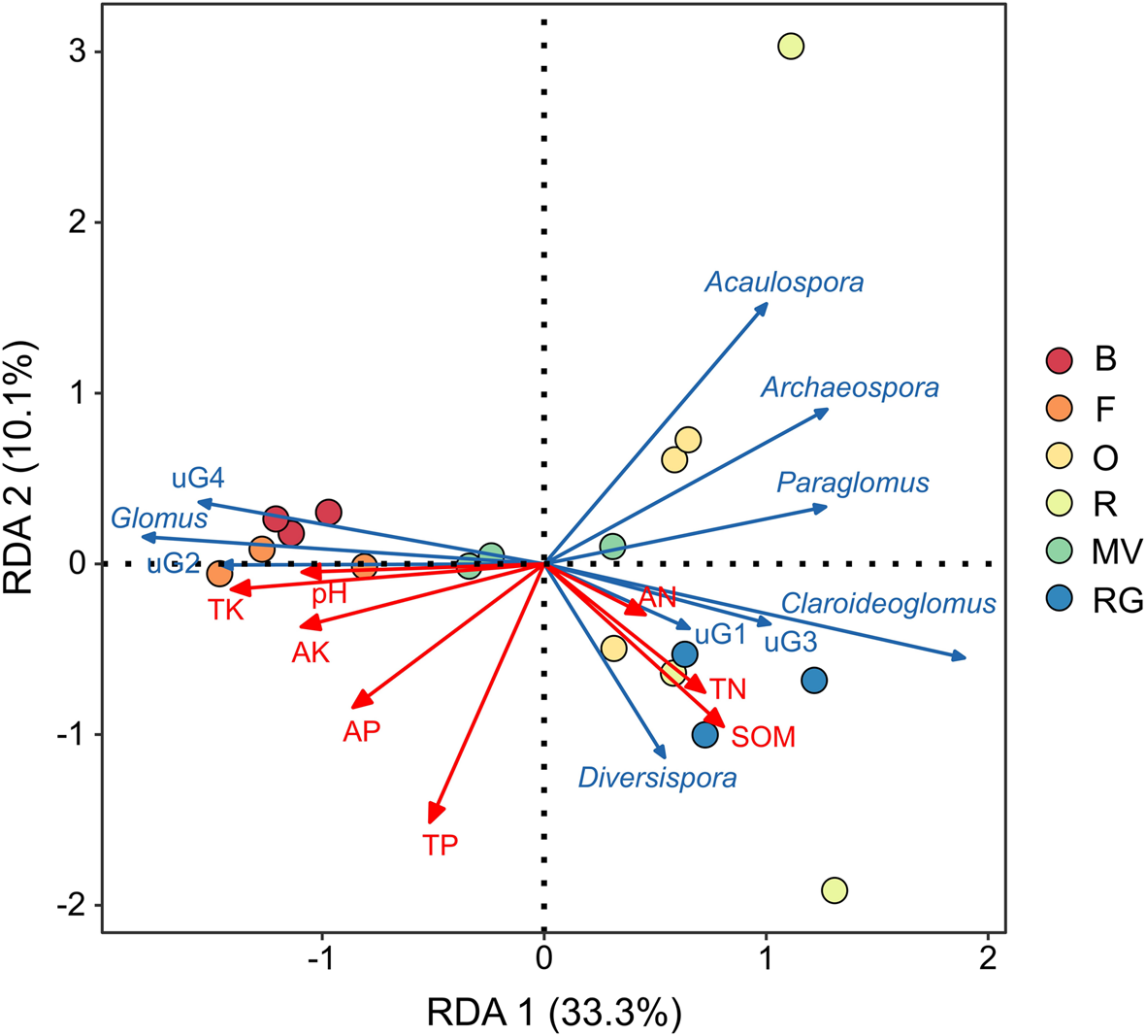
Redundancy analysis (RDA) based on the relationship between the environmental factors and AMF genera. The environmental factors are denoted by red lines with arrows, and the AMF genera are denoted by blue lines with arrows. Dots of different colors represent the different soil samples. B: background soil; F: winter fallow; O: winter oat; R: winter rye; MV: winter Chinese milk vetch; RG: winter ryegrass. uG1: *unclassified_Gigasporaceae*; uG2: *unclassified_Glomeromycetes*; uG3: *unclassified_Glomeromycota*;  uG4: *unclassified_Glomerales*.

**Fig. 6:**
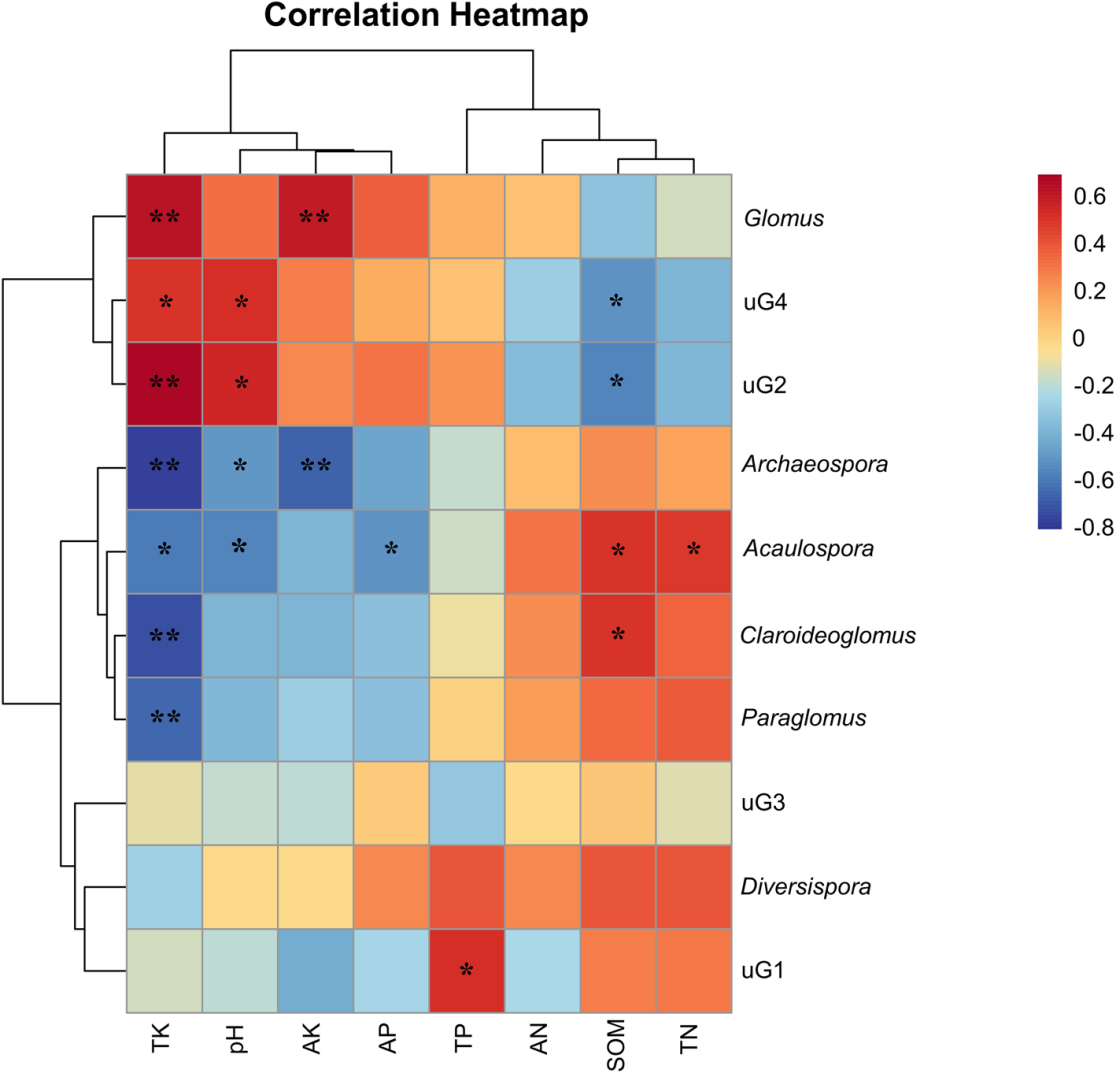
Heatmap of Spearman correlation analysis between the soil physicochemical properties and AMF genera. Significance codes: *P < 0.05; **P < 0.01. B: background soil; F: winter fallow; O: winter oat; R: winter rye; MV: winter Chinese milk vetch; RG: winter ryegrass. uG1: *unclassified_Gigasporaceae*; uG2: >*unclassified_Glomeromycetes*; uG3: *unclassified_Glomeromycota*; uG4: *unclassified_Glomerales*.

Redundancy analysis (RDA) revealed the correlation between AMF genera and environmental factors in the different treatments (Fig. 5). The first two redundancy analysis components explained 43.4% of the total variation, with RDA1 and RDA2 accounting for 33.3% and 10.1%, respectively. TK had the greatest effect on AMF genera, followed by TP and SOM. The RDA results indicated that differences in certain environmental factors regulated AMF genera. Spearman correlation analysis showed that soil TK content was significantly positively correlated with *Glomus* (*P* < 0.01) and significantly negatively correlated with *Archaeospora* (*P* < 0.01), *Claroideoglomus* (*P* < 0.01), *Paraglomus* (*P* < 0.01), and *Acaulospora* (*P* < 0.05) (Fig. 6). Soil pH was significantly and negatively correlated with *Archaeospora* and *Acaulospora* (*P* < 0.05). Soil AK was significantly negatively correlated with *Archaeospora* (*P* < 0.01) and significantly positively correlated with *Glomus* (*P* < 0.01). *Acaulospora* was also significantly positively correlated with the soil SOM and TN contents (*P* < 0.05) and significantly negatively correlated with the soil AP content (*P* < 0.05). *Claroideoglomus* was significantly and positively correlated with soil SOM content (*P* < 0.05).

### Comprehensive evaluation of effects of different winter forage crops on soil physicochemical properties and AMF community

The comprehensive evaluation of the effects of the different treatments on soil physicochemical properties, AMF communities, and forage yield are shown in Table 3. The average membership function values were ranking from high to low as follows: winter Chinese milk vetch > winter rye > winter oat = winter ryegrass > winter fallow. The average membership function value of winter Chinese milk vetch was 0.55, indicating that it had the best comprehensive effect on soil physicochemical properties, AMF community structure, diversity, and forage yield.

**Table 3:** The membership function value and rank of the comprehensive effects of different treatments on soil physicochemical properties and AMF community

Treatment	pH	SOM	TN	TP	TK	AN	AP	AK	Spore density	Chao1	Shannon	Yield	Average	Rank
F	0.66	0.29	0.25	0.70	0.94	0.26	0.78	0.66	0.06	0.32	0.42	0	0.45	4
O	0.55	0.34	0.28	0.36	0.21	0.38	0.16	0.43	0.95	0.81	0.61	0.44	0.46	3
R	0.15	0.59	0.64	0.51	0.47	0.79	0.73	0.42	0.44	0.57	0.77	0.03	0.51	2
MV	0.26	0.68	0.74	0.84	0.54	0.59	0.67	0.35	0.40	0.52	0.72	0.27	0.55	1
RG	0.26	0.37	0.28	0.74	0.56	0.41	0.81	0.16	0.51	0.25	0.19	0.93	0.46	3

O: winter oat, R: winter rye, MV: winter Chinese milk vetch, RG: winter ryegrass

## Discussion

### Winter forage crops improve soil nutrient cycling in paddy fields

Crop rotation is considered a sustainable agricultural strategy for improving soil ecological functions and crop yields (Zhao et al. 2020; Liu et al. 2023). Crop rotation also increases soil carbon and nitrogen contents and reduces fertilizer inputs for agricultural production (Cernay et al. 2018; Li et al. 2019a). Winter forage crops in paddy fields affect the physical and chemical properties of the soil. Our results showed that winter forage crops decreased soil pH, which may be due to the roots secrete organic acids in the process of forage planting, thus reducing the pH of rhizosphere soil (Adeleke et al. 2017). Legume forage crops, such as Chinese milk vetch, can use nitrogenase in root nodules to fix N_2_ in the air to form NH_4_^+^, resulting in the release of H^+^ from plant roots and a reduction in rhizosphere soil pH (Jarvis and Hatch 1985).

The increase in TN and AN content in winter Chinese milk vetch may be due to symbiosis with rhizobia for biological nitrogen fixation in Chinese milk vetch, which is a leguminous plant (Li et al. 2019b). The increase in TN and AN content in winter rye soil might be due to the low nitrogen absorption and utilization of rye as a barren-tolerant crop, and planting rye could reduce soil nitrogen loss (Krueger et al. 2011; Bauer et al. 2017). The decrease in TN and AN in the winter fallow treatment might be due to the loss of gaseous nitrogen caused by nitrification and denitrification of the soil or the loss of nitrogen in farmland runoff caused by rainfall (Fu et al. 2004; Wang et al. 2019). Winter forage crops decrease the contents of AP and AK in the soil, which are consumed to sustain forage crop growth. The different degrees of reduction in AP and AK indicate differences in the absorption and utilization of nutrients by different winter forage crops. The decrease in soil AP content during winter fallow may be duec (Liu et al. 2020b). The increase in SOM content under different treatments may have been due to the decomposition of the previous rice. The increase in SOM content under the treatment with winter rye and winter Chinese milk vetch might also be due to sufficient nitrogen nutrients in the soil, which could promote the growth of soil microorganisms and the decomposition of plant residues, thus leading to the accumulation of SOM (Xie et al. 2013; Bian et al. 2024).

### Winter forage crops increased AMF spore pool in paddy soil and improved AMF community structure and diversity

Previous studies have generally considered that AMF, as aerobic fungi, rarely exists in the flooded environment of paddy fields; therefore, the importance of AMF in paddy fields has often been overlooked (Ilag et al. 1987). However, recent studies have shown that AMF spores exist in flooded paddy environments, and because rice roots secrete oxygen, they could give AMF the oxygen needed for symbiosis (Lumini et al. 2011; Li et al. 2022). Soil spore density was related to the AMF colonization rate of the plants. AMF spores are thick-walled asexual reproductive organs formed on the mycelium outside the root when the AMF develops to a certain stage (Smith and Read 2008). In natural environments, AMF can only produce spores by forming symbiotic structures with plants (Kokkoris et al. 2020). Our results showed that winter forage crops increased AMF spore density in paddy soils. Forage crops planted in paddy fields without flooding in winter can form a symbiotic structure with native AMF in the soil. After winter planting, the mycelia develop and mature to produce spores, thereby increasing the AMF spore pool in paddy fields. The AMF spore pool was the highest in the winter oat soil. A possible reason for this is that the AMF colonization rate of winter oat was the highest, and the mycelial structure was higher; therefore, the sporulation ability was stronger. The high AMF colonization rate of oat might be due to their well-developed root systems, which provide more AMF colonization sites (Ma et al. 2023). Therefore, winter forage crops in paddy fields were conducive to the enhancement of the soil AMF spore pool, and thus the AMF colonization rate of later rice increased. In conclusion, improvement of the AMF spore pool in paddy soils using winter forage crops will be beneficial for the production of post-cropping rice.

The diversity of AMF communities in the soils of different winter forage crops differed. Higher AMF diversity improves host plant resilience to the environment and improves plant community productivity (Vogelsang et al. 2006; da Silva et al. 2014). Winter oat, rye, and Chinese milk vetch improved the soil AMF community diversity. This might be due to the differences in root morphology and root exudates of different plants, which affect the recognition and infection of AMF, and thus affect the community composition and diversity of AMF (Bao et al. 2023). There are also differences in plant growth, physiological metabolism, and nutrient absorption that affect AMF diversity (Parvin et al. 2019). The growth and development stages of winter forage crops at the time of harvest would affect nutrient requirements, and thus affect the AMF species infected. Therefore, AMF community composition and diversity were affected. For example, soil AMF diversity is higher during reproductive growth than during vegetative growth (Wang et al. 2015). Winter forage crops increased the relative abundance of *Claroideoglomus* in soil. The dominant AMF genus in the soil of winter ryegrass, oat, and rye was *Claroideoglomus*, whereas the dominant AMF genera in the soil of winter Chinese milk vetch were *Glomus* and *Claroideoglomus*. Studies have shown that *Claroideoglomus* is helpful for improving the drought resistance of host plants. In the case of low rainfall in winter in southern China, the increase in *Claroideoglomus* infecting plants increases the drought resistance of plants, resulting in large spore production in the soil and an increase in relative abundance (Deng et al. 2020). *Glomus* and *rhizobium* co-exist more easily to promote the growth of legumes; therefore, *Glomus* is also the dominant genus in the soil of winter Chinese milk vetch (Franzini et al. 2010). Some studies have found that *Claroideoglomus* can improve the absorption of Ca and K, root development, and plant biomass, which are conducive to better plant growth (Jamiolkowska et al. 2020; Aguilera et al. 2022). *Claroideoglomus* has also been found to increase the tolerance of host plants to drought as well as to some heavy metals, such as cadmium and lanthanum (Deng et al. 2020; Hao et al. 2022). Therefore, an increase in the relative abundance of *Claroideoglomus* in paddy soils may be beneficial for rice growth and development. In conclusion, winter forage crops increase AMF diversity and alter the AMF community composition, which may improve the stability and productivity of paddy ecosystems.

### Winter forage crops affected AMF community diversity by influencing soil physicochemical properties

Soil physicochemical properties are important factors that affect the AMF community structure and diversity (Mbodj et al. 2018). Soil pH, moisture, SOM content, and AP content have important effects on AMF community composition and diversity (Parvin et al. 2019). Soil pH plays a key role in the AMF community, and the most suitable pH for AMF infection is around pH 6; an increase or decrease in pH inhibits AMF infection (Qin et al. 2015; Liu et al. 2021). This finding is consistent with the results of our study. Many studies have shown that AMF community composition is significantly correlated with soil AP content (Sarkodee-Addo et al. 2020; Ibne et al. 2021). Our results showed no significant correlation between soil AP content and AMF. This may be because the AP content of the soil meets the plant growth and development needs. Under these conditions, available phosphorus was not the main factor limiting AMF colonization. Because oat roots are well-developed and could provide more infection sites for AMF (Ma et al. 2023), the colonization rate of AMF was higher, which promoted its absorption of nitrogen and phosphorus nutrients from the soil; therefore, the content of nitrogen and phosphorus in the soil was low. Our results showed that most AMF genera in paddy fields were significantly correlated with total and available K contents. The extraneous mycelia of AMF can activate soil potassium by secreting organic acids and oxalic acids, so that the potassium in soil that cannot be directly absorbed by plants can be converted into AK (Rouphael et al. 2015). Recent studies have shown that AMF can promote the absorption of potassium ions and reduce the absorption of sodium ions in plant tissues, thereby improving plant tolerance to salt stress (Wu et al. 2013). Therefore, the decrease in soil TK content might be due to the activation of potassium in the soil by mycelia after AMF infection, which is then absorbed by plants. The lowest soil AK content in winter ryegrass might be due to the differences between plant species, and the absorption and utilization of soil potassium by ryegrass was more obvious (Li et al. 2016).

It has been reported that the addition of soil nitrogen and phosphorus nutrients could change the composition of AMF community and promote the formation of dominant species (Camenzind et al. 2014). Glomerales are less sensitive to soil nitrogen content, and the relative abundance of most other AMF species decreases with increasing soil nitrogen content; however, there is no significant effect on Glomerales (Egerton-Warburton et al. 2007; Camenzind et al. 2014). Therefore, the increase in soil nitrogen content due to nitrogen fixation in winter Chinese milk vetch makes *Glomus* the dominant genus in the soil.

### Comprehensive effects of winter forage crops on soil physicochemical properties and AMF community

Winter forage crops can produce green feed, increase soil nutrient content, and improve AMF communities without fertilization, making them ideal sustainable agricultural production models. The fuzzy membership function method has been widely used in the introduction and comprehensive evaluation of agronomic traits (Jin et al. 2021). Our results showed that Chinese milk vetch had the best comprehensive effects on soil physicochemical properties, AMF communities, and yield compared with other forage crops. Although winter oat had the greatest effect on improving the soil AMF community structure, they consumed a large amount of soil nutrients. The yield of winter rye was low, and although winter ryegrass had obvious advantages in terms of yield, it consumed more soil nutrients and reduced AMF community diversity. Winter Chinese milk vetch consumes less soil nutrients and could increase the soil nitrogen content owing to nitrogen fixation. In addition, the improvement in the soil AMF community structure was good. Although the forage crop yield was low, winter milk vetch had the greatest effect.

In practical production, Chinese milk vetch is widely applied to feed livestock because of its abundance as a legume crop. In addition, Chinese milk vetch is used as green manure to increase soil nutrient content and reduce fertilizer application through nitrogen fixation (Zhou et al. 2012; Yang et al. 2024). The aboveground parts of ryegrass are typically used as feed, whereas the stubble or the rest of the plant is returned to the field to decompose and increase the nutrient content of the soil (He et al. 2019b; He et al. 2020). Oat and rye are commonly used as fodder. However, little research has been conducted on their use as green manure. Therefore, Chinese milk vetch and ryegrass are considered more suitable for production owing to their diverse functions.

In post-cropping rice, increasing the AMF spore pool in the soil of winter forage crops can increase the AMF colonization rate, thus directly promoting nutrient absorption (Cao et al. 2024). AMF infection can promote the absorption and transfer of ammonium nitrogen and nitrate nitrogen, which can improve nitrogen nutrition in rice (Koegel et al. 2017). Therefore, nitrogen-fixing plants such as Chinese milk vetch can increase soil nitrogen levels, which may be more helpful for the growth of post-cropping rice. Changes in the soil AMF community can also increase post-cropping rice production. The dominant AMF genus *Claroideoglomus* in the soil of winter forage crops promotes the absorption of Ca and K by plants and improves the resistance of host plants to drought and heavy metals (Aguilera et al. 2022; Hao et al. 2022). The dominant AMF genus, *Glomus*, in winter Chinese milk vetch soil can improve rice yield and quality (Cao et al. 2024). Nutrient consumption by planting winter forage crops is gradually released by the root stubble decomposition of winter forage crops, thus reducing the impact on post-cropping rice (He et al. 2019b).

## Conclusion

In this study, the effects of forage crops on soil physicochemical properties and AMF communities in winter paddy fields in southern China were compared. The results showed that winter forage crops could improve soil physicochemical properties and AMF community structure and diversity. The fuzzy membership function was used for a comprehensive evaluation, and the results showed that winter Chinese milk vetch had the most obvious improvement in soil physicochemical properties and the AMF community. These results provide a theoretical basis for the sustainable utilization of rice paddy ecosystems and the promotion of winter agriculture in southern China.

## Data Availability

The datasets during and/or analysed during the current study available from the corresponding author on reasonable request.
